# DDR1 and MT1-MMP Expression Levels Are Determinant for Triggering BIK-Mediated Apoptosis by 3D Type I Collagen Matrix in Invasive Basal-Like Breast Carcinoma Cells

**DOI:** 10.3389/fphar.2019.00462

**Published:** 2019-05-03

**Authors:** Charles Saby, Guillaume Collin, Maha Sinane, Emilie Buache, Laurence Van Gulick, Frédéric Saltel, Erik Maquoi, Hamid Morjani

**Affiliations:** ^1^ Unité BioSpecT, EA7506, SFR CAP-Santé, UFR de Pharmacie, Université de Reims Champagne-Ardenne, Reims, France; ^2^ INSERM, UMR1053, BaRITOn Bordeaux Research in Translational Oncology, Bordeaux, France; ^3^ Unit of Cancer, Laboratory of Tumour and Developmental Biology, Groupe Interdisciplinaire de Génoprotéomique Appliqué (GIGA), University of Liège, Liège, Belgium

**Keywords:** type I collagen, MT1-MMP, DDR1, apoptosis, breast carcinoma

## Abstract

Type I collagen is the major adhesive component in breast interstitial stroma, which represents the first barrier against tumor cell invasion after basement-membrane degradation. Among cellular receptors, type I collagen is able to activate discoidin domain receptors DDR1 and DDR2. We have previously shown that in 3D collagen matrix, DDR1 plays a key role as it promotes cell growth suppression and apoptosis through the upregulation of the pro-apoptotic mediator BIK in noninvasive luminal-like breast carcinoma cells. We have also shown that MT1-MMP is able to rescue these cells and protect them against the effects induced by collagen/DDR1/BIK axis. Our data suggested that the protective effect of MT1-MMP might be mediated through the degradation of type I collagen and/or DDR1 cleavage. Decreased DDR1 expression has been associated with the epithelial to mesenchymal transition process in breast cancer, and its overexpression in aggressive basal-like breast cancer cells reduces their invasiveness in 3D cultures and *in vivo*. In the present work, we propose to study the role of MT1-MMP in the resistance against collagen-induced apoptosis in basal-like breast carcinoma MDA-MB-231 cells. We aimed to investigate whether MT1-MMP depletion is able to restore apoptosis mediated by collagen/DDR1/BIK axis and to verify if such depletion is able to restore full-length DDR1 expression and phosphorylation. ShRNA strategy against MT1-MMP mRNA was able to partially restore full length DDR1 expression and phosphorylation. This was accompanied by a decrease in cell growth and an upregulation of BIK expression. This suggested that MT1-MMP expression in basal-like breast carcinoma cells, in addition to a low basal level of DDR1 expression, protects these cells against collagen-induced apoptosis *via* DDR1 cleavage. Since DDR1 was moderately expressed in MDA-MB-231 cells, we then investigated whether overexpression of DDR1 could be able to increase its ability to suppress cell growth and to induce apoptosis. Data showed that overexpression of DDR1 induced a decrease in cell growth and an increase in BIK expression, suggesting that moderate expression level of full length DDR1 in basal-like breast carcinoma provides them with a capacity to resist to collagen-induced cell growth suppression and apoptosis. Finally, the combined overexpression of DDR1 and depletion of MT1-MMP in MDA-MB-231 cells synergistically increased collagen-induced cell growth suppression and apoptosis to a level similar to that observed in luminal breast carcinoma. Taken together, our data suggest that during the acquisition of mesenchymal features, the low level of DDR1 expression should be considered as an important biomarker in the prognosis of basal-like breast carcinoma, conferring them a high rate of cell growth and resistance to BIK-mediated apoptosis induced by the stromal collagen.

## Introduction

The extracellular matrix plays an important role in the regulation of tumor progression (Pickup et al., 2014). Type I collagen is the major adhesive component in breast tumors and represents the first barrier against tumor cells after basement-membrane degradation (Mouw et al., 2014). Among cellular receptors, type I collagen is able to activate integrins (α1β1, α2β1, α10β1, and α11β1) (Humphries et al., 2006; Leitinger, 2011) and discoidin domain receptors DDR1 and DDR2 (Vogel et al., 1997; Leitinger, 2011; Carafoli and Hohenester, 2013; Fu et al., 2013b). DDRs belong to the receptor tyrosine kinase family and play important roles in physiological and pathological conditions (Borza and Pozzi, 2013; Leitinger, 2014).

DDR1 and DDR2 have been demonstrated to predominantly regulate tumor progression (Valiathan et al., 2012). In addition to their oncogenic function, it has also been reported that these receptors can play a role of tumor suppressor (Rammal et al., 2016; Henriet et al., 2018). For example, in 3D collagen matrix, DDR2 is able to suppress tumor cell growth (Wall et al., 2005; Iwai et al., 2013; Saby et al., 2016; Terashima et al., 2016). DDR1 is also a key factor in collagen-induced apoptosis in noninvasive luminal-like breast carcinoma cells. In this case, apoptosis is mostly characterized by the expression of the pro-apoptotic mediator BIK (Assent et al., 2015; Saby et al., 2018). In addition to a high level of DDR1 expression, the luminal-like breast carcinoma cells are characterized by a low expression level of MT1-MMP (Maquoi et al., 2012). However, in invasive basal-like breast carcinoma cells, DDR1 has been described to promote linear invadosomes and tumor invasion (Juin et al., 2014). At the opposite of the luminal-like breast carcinoma cells, the basal-like ones express low levels of DDR1 and a high level of MT1-MMP (Maquoi et al., 2012; Croissant et al., 2018). In some of the basal-like breast carcinoma cells, it has been reported that the low level of DDR1 expression could be compensated by an increase in DDR2 expression (Toy et al., 2015). This low level of DDR1 is yet sufficient to promote tumor invasion in a kinase-independent manner (Juin et al., 2014). Interestingly, studies have shown that a low DDR1 expression is correlated with poor relapse-free survival, confirming its controversial role in tumor progression (Ford et al., 2007; Takai et al., 2018). Concerning the role of MT1-MMP in the regulation of collagen-induced apoptosis, we and others have previously shown that when expressed by luminal-like breast carcinoma, this metalloproteinase protects cells against collagen-induced apoptosis (Maquoi et al., 2012; Albrechtsen et al., 2013; Saby et al., 2018). These studies supported a model in which MT1-MMP inactivates the collagen/DDR1/BIK apoptosis signaling pathway through the degradation of collagen (Amar et al., 2017) and/or the cleavage of DDR1 (Fu et al., 2013a).

Basal-like breast cancers are among the most aggressive and deadly breast cancer subtypes, displaying a high metastatic ability associated with mesenchymal features. These mesenchymal features are acquired as a consequence of an epithelial to mesenchymal transition (EMT). This process is classically characterized by the dedifferentiation from an epithelial to mesenchymal phenotype, marked by the decreased expression of E-cadherin and increased expression of vimentin, as well as expression of cellular proteases such as MT1-MMP. Decreased DDR1 expression has been associated with the EMT process in breast cancer, and its overexpression in aggressive basal-like breast cancer cells reduces their invasiveness in 3D cultures and *in vivo*, supporting an anti-migratory function of DDR1 in this cancer (Koh et al., 2015). In a previous study, we have examined this correlation in multiple breast cancer cell lines, by analyzing expression levels of E-cadherin, vimentin, DDR1, DDR2, MT1-MMP, and BIK mRNAs in 58 breast cancer cell lines (Saby et al., 2018). High DDR1 expression was clearly observed in the E-cadherin-high and vimentin-low epithelial cell lines. In contrast, the more mesenchymal breast cancer cell lines (E-cadherin-low and vimentin-high) were characterized by a weak DDR1 level, a high MT1-MMP, and a low BIK expression (Saby et al., 2018). We have then suggested that the acquisition of mesenchymal features including the downregulation of DDR1 and the overexpression of collagenolytic proteinases such as MT1-MMP provide breast carcinoma cells with an increased capacity to resist to apoptosis induced by 3D collagen matrix. More recently, DDR1 ablation *in vivo* was reported to confer a basal-like phenotype to luminal-like breast carcinoma population and to increase their metastatic potential (Takai et al., 2018).

Treatment of the basal-like breast carcinoma MDA-MB-231 cells with BB-94, a synthetic broad spectrum MMP inhibitor, was shown to restore a collagen-induced apoptosis (Maquoi et al., 2012). Likewise, a specific depletion of MT1-MMP using a siRNA strategy increased the number of apoptotic bodies in these cells. However, the potential contribution of the collagen/DDR1/BIK axis was not investigated (Albrechtsen et al., 2013).

In the present work, we aim at studying the contribution of MT1-MMP in the resistance of basal-like breast carcinoma cells against collagen-induced apoptosis. Whether MT1-MMP silencing is able to restore apoptosis induced through the collagen/DDR1/BIK axis, as well as to restore full length DDR1 expression and phosphorylation, will be investigated. Since DDR1 is moderately expressed in basal-like breast carcinoma cells, we propose to explore whether overexpression of DDR1 could restore apoptosis. Finally, we will test whether the simultaneous silencing of MT1-MMP and overexpression of DDR1 in basal-like breast carcinoma cells are able to restore apoptosis to a level similar to that observed in luminal-like breast carcinoma cells. Our data suggest that, in addition to the known markers related to mesenchymal features (basal-like), the concomitant overexpression of MT1-MMP and downregulation of DDR1 expression should be considered as important biomarkers in the prognosis of breast carcinomas.

## Materials and Methods

### Cell Culture

The human breast adenocarcinoma cell lines MCF-7 (HTB-22) and MDA-MB-231 (HTB-26) were purchased from the American Type Culture Collection (ATCC). MCF-7 cells stably transfected with the full-length MT1-MMP vector (MCF-7 MT1-MMP) and MCF-7 cells transfected with the empty vector (MCF-7 VEC) were obtained as previously described (Maquoi et al., 2012). MCF-7 and MDA-MB-231 cell lines were cultured in DMEM (4,5 g/l glucose) with Glutamax I (PAN-Biotech, p04-04500) supplemented with 10% fetal bovine serum (Dominique Dutscher, S1810-500) and 1% penicillin-streptomycin (Invitrogen, 15140). Cultures were maintained at 37°C in a humidified atmosphere containing 5% CO_2_ (v/v). Cells were routinely passaged at preconfluency using 0.05% trypsin, 0.53 mM EDTA (Invitrogen, 25300) and screened for the absence of mycoplasma using PCR methods.

### Preparation and Characterization of Type I Collagen

Fibrillar native type I collagen was extracted from tail tendons of 2-month-old rats and prepared as already described (Garnotel et al., 2000). Briefly, type I collagen was extracted from tail tendons of Wistar rats (Janvier) using 0.5-M acetic acid at 4°C, in the presence of protease inhibitors. Then, type I collagen was specifically precipitated with NaCl 0.7 M and centrifuged. The precipitate was then re-suspended in 18 mM acetic acid, and salts used during the precipitation step were eliminated by dialysis against distilled water for 1 week at 4°C. Finally, the collagen was characterized as described in our previous work, before use (Saby et al., 2016, 2018).

### Plastic and 3D Cell Culture

Type I collagen effect on breast adenocarcinoma cells growth was studied in 24-well plates. For plastic condition, cells were seeded at a density of 3 × 10^4^ cells/well (1 ml/well). For 3D cell culture, 3 × 10^4^ cells were resuspended in 100-μl fetal bovine serum and mixed with a solution containing 100 μl of 10X culture medium DMEM (Gibco, 52100), 100 μl NaHCO_3_ (0.44 M), 100 μl H_2_O, 90 μl NaOH 0.1 M, 10 μl glutamine 200 mM and 500 μl collagen 3 mg/ml. Then, 1 ml/well of this solution was deposited in 24-well plates, and gels were polymerized at 37°C during 30 min. Finally, 1 ml of complete culture medium was added on top of each gel and the plates were incubated at 37°C. After 5 days, the covering medium was removed, and cell populated gels were digested with collagenase P (2 mg/ml – Roche, 11213873001). Viability and cellular density of this suspension were determined by phase contrast microscopy using Kova^®^ Glasstic^®^ Slides (Kova International Inc, 87144). In some 3D culture experiments, cells were treated with DDR1 pharmacological inhibitors nilotinib (Selleckchem, No.S1033), at 100 nM.

### Vectors, Transfection, and Infection

DDR1-GFP overexpression was performed with pLVX-CMV-DDR1-GFP. DDR1-GFP lentiviral particles were generated through co-transfection of 293 T cells with pCMV ΔR8.91 (gag-pol) and phCMVG-VSVG (env) expression constructs using the FuGene 6 transfection reagent (Promega) according to the manufacturer’s recommendations. Three days after transfection, the viral supernatant mixed with fresh medium (1 of 4) and hexadimethrine bromide at 8μg/ml (Sigma) was used to infect MDA-MB-231 cells. Infected cells were selected using puromycin (Invivogen) at 1 μg/ml.

MT1-MMP knock-down was achieved with MMP-14-specific shRNAs (Buache et al., 2014). shRNA MMP-14 retroviral particles were generated through co-transfection of 293 T cells with pCL-Ampho expression constructs using the FuGene 6 transfection reagent (Promega) according to the manufacturer’s recommendations. Three days after transfection, the viral supernatant mixed with fresh medium (1 of 4) and hexadimethrine bromide at 8μg/ml (Sigma) was used to infect MDA-MB-231 cells. Infected cells were selected using puromycin (Invivogen) at 1 μg/ml.

### Reverse Transcription and Real-Time Quantitative PCR

Total RNA was isolated with a phenol-chloroform extraction method, using RNA Extracol (EURx). Then, 1 μg of total RNA was converted to cDNA by reverse transcription using the Maxima First Strand cDNA Synthesis kit (Thermo Fisher Scientific) according to the manufacturer’s recommendations. Real-time PCR was performed using a Maxima SYBR GREEN/ROX qPCR Master Mix (Thermo Fisher Scientific, #KO222) on the Stratagene Mx3005P qPCR detection system (Agilent Technologies). Polymerase chain reaction conditions were 15min at +95°C, followed by 35 cycles each consisting of 15s at +95°C (denaturation) and 30s at +60°C (annealing/extension). Results were standardized to the eEF1a1 gene expression by calculating ΔCt using the formula ΔCt = (Ct gene of interest – Ct eEF1A). Gene expression was represented as 2-ΔCt. The following primers were used: BIK forward primer: 5′-aggacctggaccctatggag-3′ and reverse primer: 5′-ccctgatgtcctcagttggg-3′; eEF1A1 forward primer: 5′-ctggagccaagtgctaacatgcc-3′ and reverse primer: 5′-ccgggtttgagaacaccagtc-3′.

### Quantification of Annexin V Positive Cells

Cells were cultured in type I collagen 3D matrices as described in “plastic and 3D cell culture”. After 36 hours, 1 × 10^5^ cells were harvested for each condition using collagenase P at 2 mg/ml, washed twice with PBS and analyzed using the Muse^®^ Annexin V and Dead Cell Assay Kit, Millipore, MCH100105, according to the manufacturer’s instructions.

### Western Blotting

Cells were cultured in type I collagen 3D matrices or plastic as described in the “plastic and 3D cell culture”. After 5 days, cells were harvested using collagenase P at 2 mg/ml for 3D matrices, washed twice with PBS, and lysed with RadioImmuno Precipitation Assay (RIPA) buffer (Thermo Fisher Scientific, 89900), supplemented with Halt™ Protease and Phosphatase Inhibitor Cocktail 1X (Thermo Scientific, 78442). For plastic conditions, cells were directly washed and lysed without the use of collagenase P. Cell lysates were sonicated and clarified by centrifugation at 14 000 × g at 4°C for 15 min. Then, total protein content was estimated by bicinchoninic acid (BCA) assay method (Thermo Scientific, 23227), and proteins were separated by SDS-PAGE and transferred to a nitrocellulose membrane. Membranes were blocked with Tris Buffered Saline (TBS) (0.02 M Tris–HCl, 0.137 M NaCl, pH 7.6) containing 0.1% Tween (TBS-T) and 5% Bovine Serum Albumin (BSA) at room temperature during 1 hour and incubated overnight at 4°C with anti-DDR1 (Cell Signaling Technology, #5583), anti-phospho DDR1 (Tyr 792) (Cell Signaling Technology, #11994), anti-GAPDH antibodies (Cell Signaling Technology, #5174), and anti-MT1-MMP, obtained from Dr. Tomasetto C. L (IGBMC, Illkirch, France) (Buache et al., 2014). Membranes were washed with TBS-T and incubated with peroxidase conjugated anti-rabbit secondary antibody (Cell Signaling Technology, #7074) at room temperature for 1 h. Chemiluminescent detection was performed by using an ECL Prime Kit (GE Healthcare, RPN2236). Electrophoretic images were analyzed with ImageJ software.

### Expression Level of EMT Marker in Breast Cancer Cell Lines

The expression levels of E-cadherin, vimentin, DDR1, DDR2, α2 integrin, α11 integrin, COL1A1, MT1-MMP, BIK, estrogen receptor α, progesterone receptor, and HER2 mRNAs in 58 breast cancer cell lines were obtained by interrogating the Broad-Novartis Cancer Cell Line Encyclopedia (CCLE) database.

### Correlation Analyses Between DDR1 and BIK mRNA Levels in Breast Cancer

Correlations analysis between DDR1 and BIK mRNA levels were performed by interrogating gene expression data sets contained at cBioPortal^1^ and Breast Cancer Gene-Expression Miner (bc-GenExMiner)^2^. bc-GenExMiner contains 36 datasets including 5861 breast cancer patients (Jézéquel et al., 2012). cBioPortal was used to explore the TCGA breast cohort (Ciriello et al., 2015).

### KM Plotter Database Analysis

Kaplan–Meier curves were generated with the Kaplan–Meier plotter website (http://kmplot.com), using a database of public microarray datasets (Györffy et al., 2010). Automatic cut-off scores were selected during queries, and relapse-free survival (RFS) was selected. Number of cases, hazard ratios (HRs), 95% confidence intervals, and *p*-values were extracted from the KM plotter webpage. The analyses were performed with the mean expression of the 4 DDR1 Affymetrix probes (1007_s_at, 207169_x_at, 208779_x_at, and 210749_x_at).

### Statistical Analyses

Data are presented as mean ± standard error of the mean (SEM) from three independent experiments. Statistical significance was assessed with Student’s *t* test, or with one-way ANOVA, followed by Tukey’s multiple comparison test. *p* < 0.05 was considered as significant (*p < 0.05; **p < 0.01; ***p < 0.001).

## Results

### MCF-7 and MDA-MB-231 Cells Present Different Expression Profiles for EMT Markers

**Figure 1:**
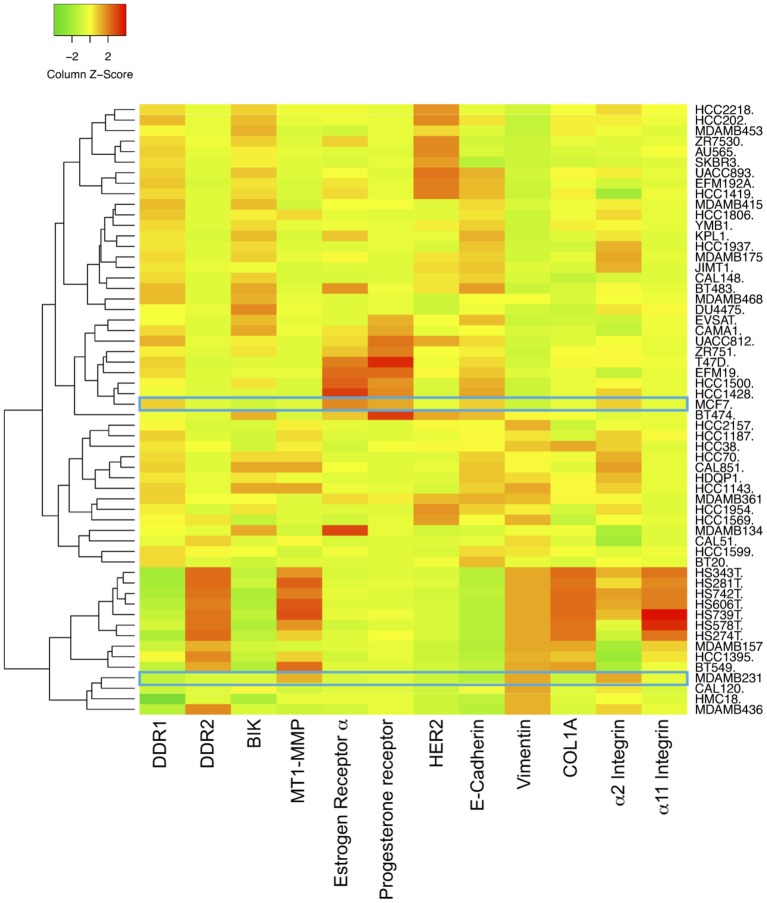
Expression of DDR1, BIK and MT1-MMP in basal and luminal breast cancer cell lines. Heat map depicting the relative expression of E-cadherin (CDH1, luminal marker), vimentin (VIM, basal marker), DDR1, DDR2, α2 integrin, α11 integrin, αv integrin, COL1A1, MT1-MMP, and BIK in 58 breast cancer cell lines.

The human breast adenocarcinoma cell lines MCF-7 and MDA-MB-231 cells display different phenotypes. While the noninvasive luminal-like MCF-7 cells present typical epithelial features, the invasive basal-like MDA-MB-231 cells present a more mesenchymal phenotype (Maquoi et al., 2012). The expression levels of E-cadherin, vimentin, DDR1, DDR2, α2 integrin, α11 integrin, COL1A1, MT1-MMP, BIK, estrogen receptor α, progesterone receptor, and HER2 mRNAs in 58 breast cancer cell lines were analyzed *in silico*, by using the Broad-Novartis Cancer Cell Line Encyclopedia (CCLE) database. As shown in Figure 1, the 58 breast cancer cell lines segregate into two distinct groups. The first one includes cells with a relatively high level of E-cadherin, DDR1, BIK, and a low level of MT1-MMP, vimentin, DDR2, α2 integrin, α11 integrin, and COL1A1 and is essentially composed of cells with an epithelial phenotype like the MCF-7 cells, which are estrogen receptor α and progesterone receptor positive. The second group is represented by cells with a low level of E-cadherin, DDR1, and BIK and a high level of MT1-MMP, vimentin, and other markers. This group is composed by cells with mesenchymal features like the MDA-MB-231 triple negative cells. These data show that in addition to the switch of classical EMT markers expression, there is a concomitant switch of DDR1 and BIK expression between the epithelial and mesenchymal breast cancer cells.

### DDR1 Is More Expressed in Luminal Breast Adenocarcinomas Than in the Basal Ones

**Figure 2:**
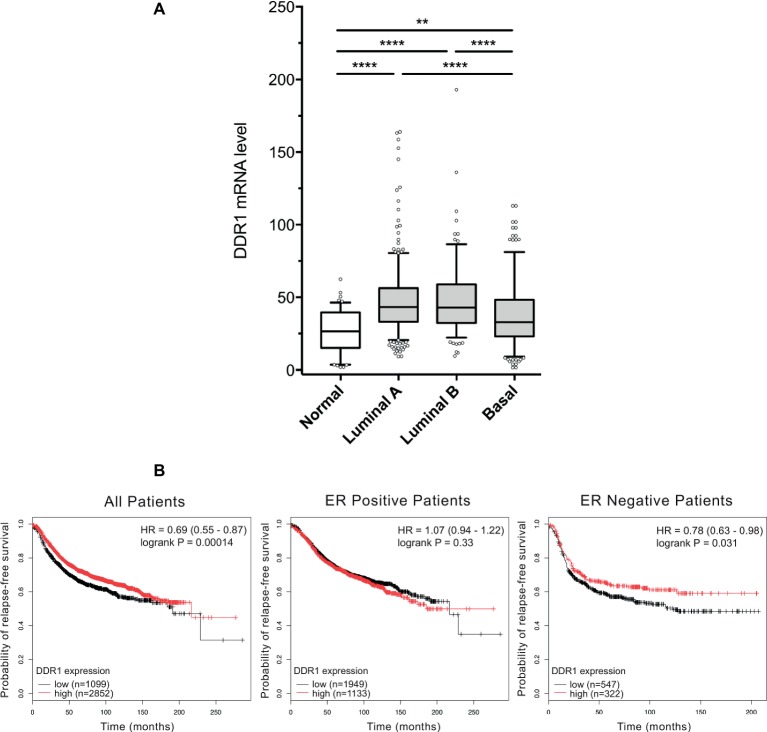
DDR1 expression in breast cancer and the associated correlation with prognosis in ER negative tumors. **(A)** Box-and-whisker plots comparing the expression level of DDR1 among normal, luminal A, luminal B, and basal subtypes. Shaded rectangles represent interquartile range; line in the middle of each rectangle represents median value. Lines extending from the interquartile range mark the 5th and 95th percentile values, and the individual open circles represent values that are either above the 95th percentile or below the 5th percentile for each distribution. **, *p* < 0.01; ****, *p* < 0.0001. **(B)** Kaplan–Meier representation of relapse-free survival (RFS) probability over time for breast cancer patients irrespective of the ER status, ER positive and ER negative patients with high or low DDR1 expression.

DDR1 expression level was analyzed in a cohort of TCGA breast cancer patients. As shown in Figure 2A, basal-like breast tumors express significantly lower levels of DDR1 mRNA when compared to luminal A and B tumors. DDR1 expression was then studied in a cohort of 3,951 breast tumors from patients where relapse free survival (RFS) was assessed. As shown in Figure 2B, high DDR1 expression is associated with a better RFS probability in the total patient population (HR = 0.69, *p* = 0.00014) and in estrogen receptor negative tumors (HR = 0.78, *p* = 0.031) but not in the estrogen receptor positive tumors (HR = 1.07, *p* = 0.33). In contrast, DDR1 expression level was not associated with an altered RFS probability when patients were stratified according to the expression of progesterone receptor, HER2, tumor grades, or intrinsic subtypes (data not shown).

### BIK and DDR1 mRNA Levels Are Positively Correlated in Basal-Like Breast Tumors

**Figure 3:**
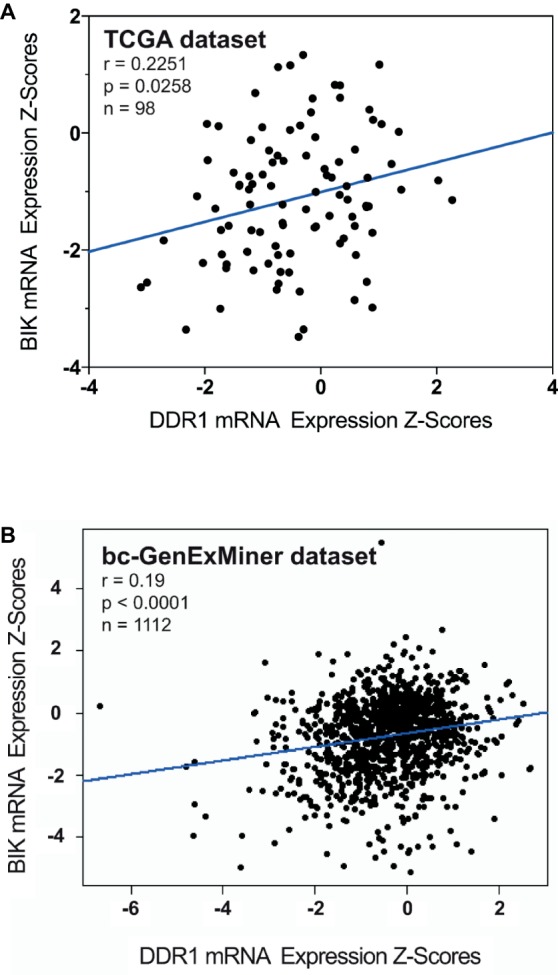
Correlation between BIK and DDR1 mRNA expression levels in breast cancer patients. **(A)** from the TCGA dataset; and **(B)** from the bc-GenExMiner dataset.

Correlation between BIK and DDR1 mRNA expression levels was analyzed using published genomic data from two dataset of breast cancer patients, the TCGA and the bc-GenExMiner (Jézéquel et al., 2012). As shown in Figure 3A, the expression level of apoptosis marker BIK was positively correlated to that of DDR1 in basal-like breast tumors (r = 0.2251, *p* = 0.0258). The same results were obtained with the bc-GenExMiner dataset that shows a positive correlation between BIK and DDR1 mRNA expression (r = 0.19, *p* < 0.0001; Figure 3B).

### MCF-7 and MDA-MB-231 Cells Express Different Levels of DDR1 and MT1-MMP

**Figure 4:**
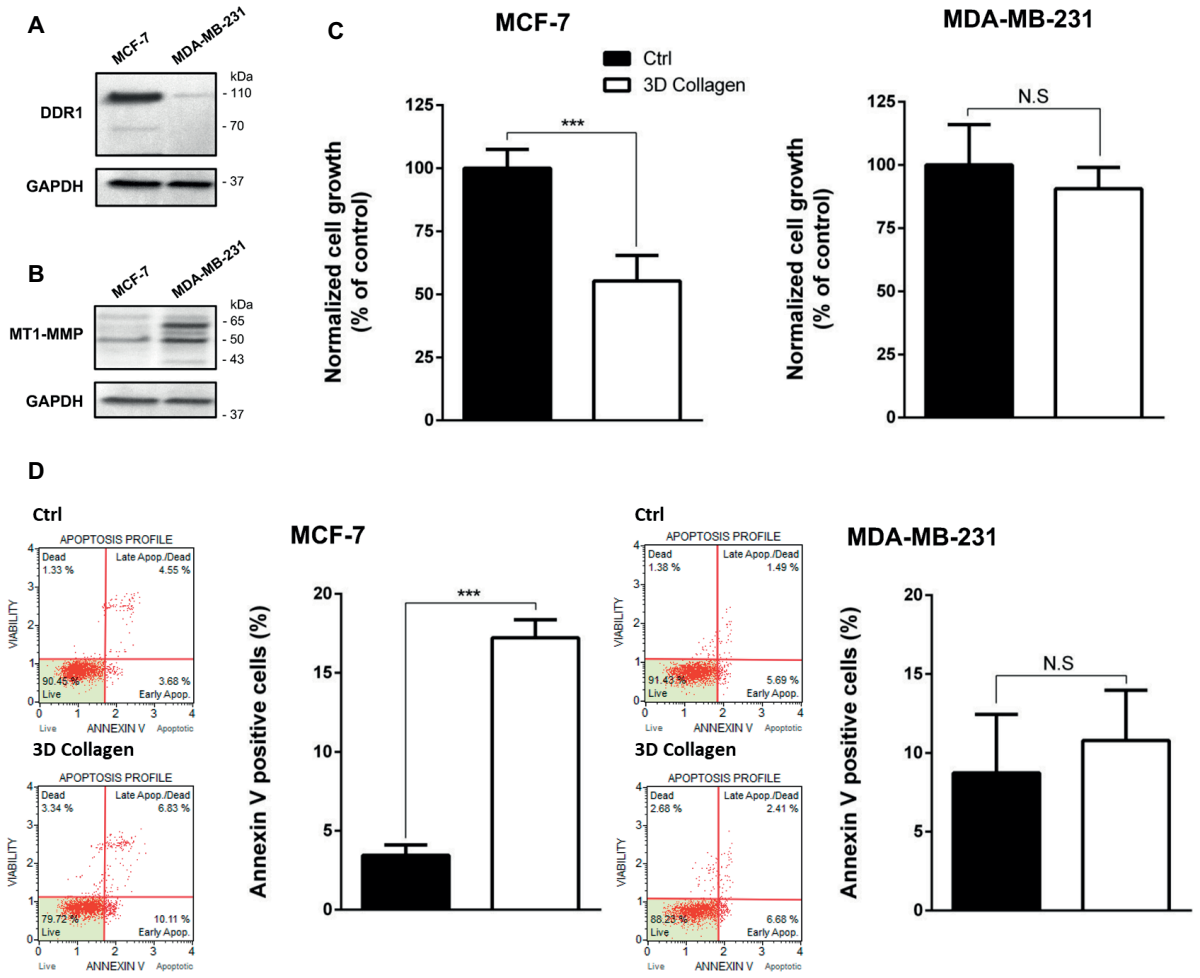
Effect of type I collagen on the growth of MCF-7 luminal breast cancer cells and MDA-MB-231 basal-like breast cancer cells. **(A)** DDR1 protein expression was evaluated using Western Blotting. GAPDH was used as a loading control. **(B)** MT1-MMP protein expression was evaluated using Western Blotting. GAPDH was used as a loading control. **(C,D)** MCF7 (left panel) and MDA-MB-231 (right panel) cells were seeded on plastic or in 3D type I collagen matrices. After 5 days of culture, viable cell density was evaluated by phase contrast microscopy **(C)** and after 36 h of culture, apoptosis was quantified using the Muse^®^ annexin V and dead cell assay kit **(D)**. Values represent the mean ± S.D. of three independent experiments (***p < 0.001, N.S. = not significant).

DDR1 has the unique ability among the tyrosine kinase receptors to be activated only by type I collagen in its fibrillary state. We have previously shown that DDR1 was able to initiate apoptosis in luminal-like breast carcinoma MCF-7 cells embedded in 3D type I collagen matrices (Assent et al., 2015; Saby et al., 2018). We have also shown that MT1-MMP, the primary enzyme used by stromal and cancer cells to cleave and migrate through fibrillar collagen, plays a crucial role in the regulation of cell growth and survival in 3D type I collagen matrices. Here, DDR1 and MT1-MMP protein expression levels were compared by western blot analysis in MDA-MB-231 and MCF-7 cells. In agreement with *in silico* transcriptomic data (Figure 1), MCF-7 cells express a high level of DDR1 and a low level of MT1-MMP, whereas an opposite expression profile was observed in MDA-MB-231 cells (Figures 4A,B).

### 3D Type I Collagen Matrices Promote Apoptosis in MCF-7 Cells

Since 3D type I collagen matrices are known to induce apoptosis in luminal-like breast carcinoma cell lines, thus regulating tumor cell growth, we first compared the growth of MCF-7 and MDA-MB-231 cell lines, cultivated for 5 days in two different experimental settings: (i) plated on plastic (used as a control) or (ii) suspended within a 3D native type I collagen gel. MCF-7 cells exhibited a significantly lower cell growth in 3D collagen when compared to 2D plastic condition, whereas MDA-MB-231 cells showed a similar cell growth in the two culture conditions (Figure 4C). Since it has been shown that type I collagen/DDR1 axis was able to induce apoptosis in MCF-7 cells (Assent et al., 2015; Saby et al., 2018), we then compared apoptosis between MCF-7 and MDA-MB-231 cells. As shown in Figure 4D, 3D collagen strongly increased apoptosis in MCF-7 cells as evidenced by annexin V staining, whereas it failed to modulate apoptosis in MDA-MB-231 cells. These data demonstrate that the luminal and basal-like breast carcinoma cell lines exhibit a different behavior in a 3D type I collagen environment.

### MT1-MMP Overexpression in MCF-7 Cells Induces a Cleavage of DDR1 and Promotes Cell Growth

**Figure 5:**
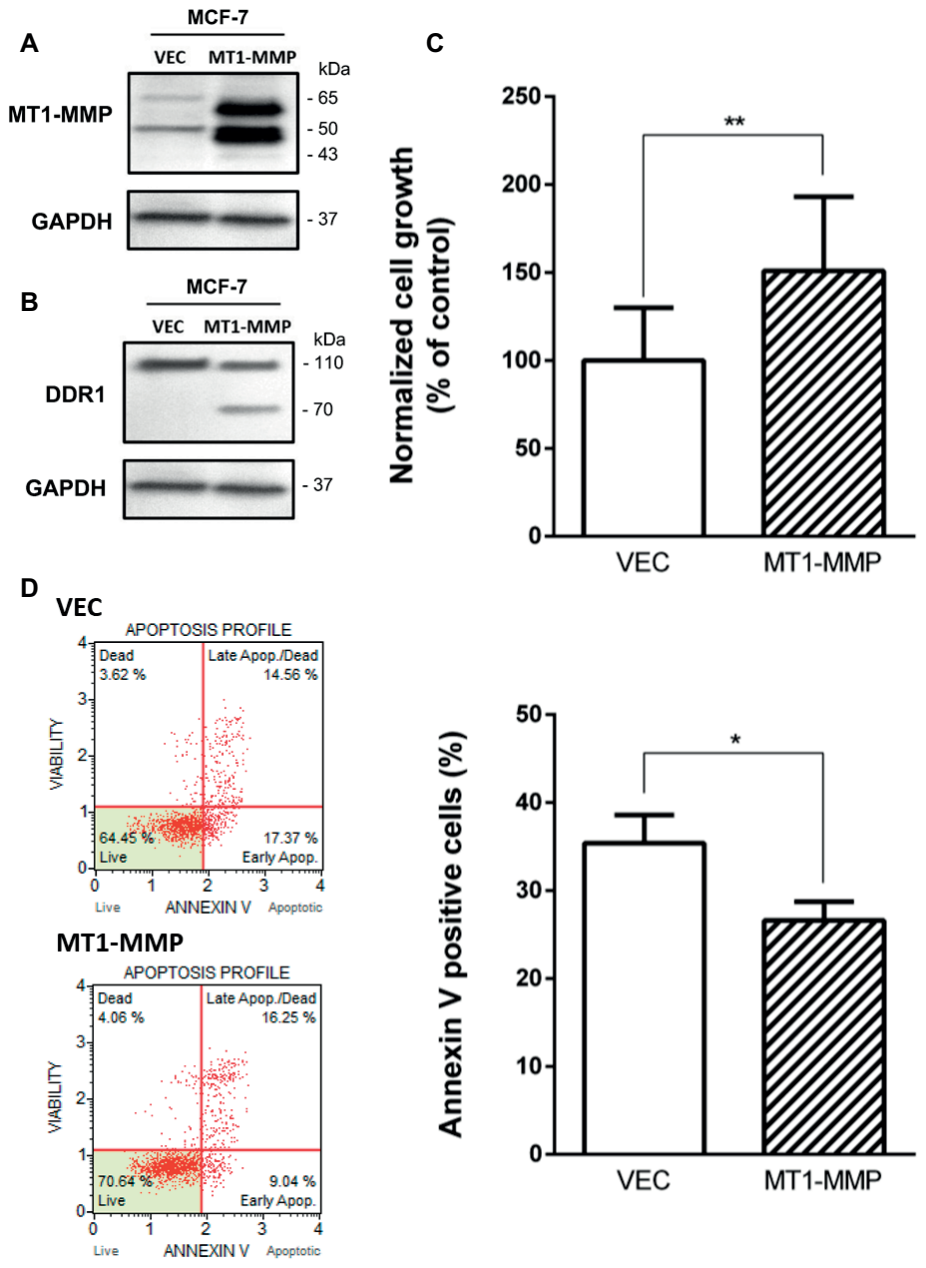
Effect of MT1-MMP expression on type I collagen-induced growth reduction and apoptosis in MCF-7. **(A)** Western blot analysis of ectopically expressed MT1-MMP in MCF-7. GAPDH was used as a loading control. **(B)** Western blot analysis showing the cleavage of DDR1 in MCF7 ectopically expressing MT1-MMP. GAPDH was used as a loading control. **(C,D)** MCF-7 VEC (empty vector) and MCF-7 MT1-MMP (full length MT1-MMP expression vector) cells were seeded in type I collagen 3D matrices. After 5 days of culture, viable cell density was evaluated by phase contrast microscopy **(C)** and after 36 h of culture, apoptosis was quantified using the Muse^®^ annexin V and dead cell assay kit **(D)**. Values represent the mean ± S.D. of three independent experiments (*p < 0.05, **p < 0.01).

Previous works have shown that MT1-MMP was able to cleave DDR1 (Fu et al., 2013a; Assent et al., 2015), and that depletion of MT1-MMP was able to increase apoptosis in basal-like breast cancer cells (Albrechtsen et al., 2013). Here, we investigated whether MT1-MMP is involved in the cleavage of DDR1 providing cell protection against the cell growth suppressor and pro-apoptotic effects of type I collagen/DDR1/BIK signaling pathways. To this end, MCF-7 cells that are lacking endogenous MT1-MMP were transfected with a full-length MT1-MMP expression vector (MCF-7 MT1-MMP) or an empty vector (MCF-7 VEC), as already described (Maquoi et al., 2012; Saby et al., 2018). In MCF-7 MT1-MMP cells, MT1-MMP is expressed as a 60-kDa mature form and a 43-kDa autoproteolytic degradation product (Figure 5A). In those cells, DDR1 expression was analyzed by western blotting. As shown in Figure 5B, MCF-7 MT1-MMP cells present both the full length DDR1 and a lower molecular weight form which corresponds to a cleaved membrane-anchored c-terminal fragment (Fu et al., 2013a), whereas MCF-7 VEC cells present only the full length DDR1. These data confirmed the previously reported efficient cleavage of DDR1 by MT1-MMP (Fu et al., 2013a; Assent et al., 2015). We then studied the effect of MT1-MMP overexpression on MCF-7 cells growth and apoptosis. As shown in Figures 5C,D, MCF-7 MT1-MMP exhibited a higher cell growth and a lower apoptosis rate.

### MT1-MMP Depletion in MDA-MB-231 Cells Induces an Increase in Full Length DDR1 Expression and Promotes Cell Apoptosis

**Figure 6:**
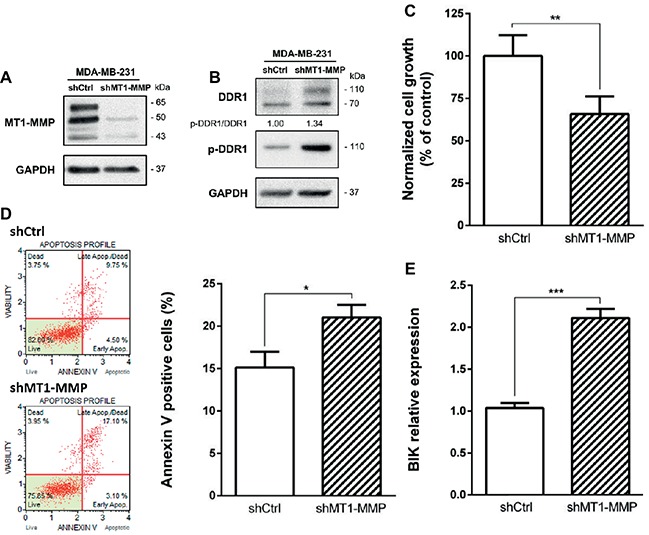
Effect of MT1-MMP silencing in MDA-MB-231 on cell growth and survival in 3D type I collagen matrices. **(A)** Western blot analysis confirming efficient knock down of MT1-MMP in MDA-MB-231 shMT1-MMP. GAPDH was used as a loading control. **(B–E)** MDA-MB-231 shCtrl and MDA-MB-231 shMT1-MMP cells were embedded in 3D type I collagen matrices. After 36 h of culture, cell extracts were analyzed for tyrosine phosphorylation of DDR1 and total DDR1 by Western blotting. GAPDH was used as a loading control **(B)**. After 5 days of culture, viable cell density was evaluated by phase contrast microscopy **(C)**. After 36 h of culture, apoptosis was quantified using the Muse^®^ annexin V and dead cell assay kit **(D)** and BIK expression was measured by RT-PCR **(E)**. Values represent the mean ± S.D. of three independent experiments (*p < 0.05; **p < 0.01; ***p < 0.001).

Since MT1-MMP is involved in DDR1 cleavage, partially protecting cells from the type I collagen induced apoptosis observed in the luminal breast cancer cell line MCF-7, we then investigated the effect of MT1-MMP depletion on DDR1 expression profile in the basal-like cell line MDA-MB-231. Albrechtsen et al. showed that the depletion of MT1-MMP using a siRNA strategy in MDA-MB-231 cells was able to increase the number of apoptotic bodies. However, the potential implication of the collagen/DDR1/BIK axis in this apoptotic process was not characterized (Albrechtsen et al., 2013). To that purpose, MDA-MB-231 cells were stably transfected with a shRNA directed against MT1-MMP. As shown in Figure 6A, MDA-MB-231 shMT1-MMP cells exhibit a lower level of MT1-MMP when compared to shCtrl transfected cells. By using western blotting, we then compared DDR1 expression and phosphorylation in shCtrl and shMT1-MMP MDA-MB-231 cells. Figure 6B shows that the full length DDR1 is more expressed in MDA-MB-231 shMT1-MMP cells than in MDA-MB-231 shCtrl cells. Also, the analysis of DDR1 phosphorylation in both conditions shows a 1.34-fold increase in pDDR1/DDR1 ratio in MDA-MB-231 shMT1-MMP cells compared to shCtrl cells. We then analyzed cell growth and apoptosis in both cell lines. As shown in Figure 6C, MDA-MB-231 shMT1-MMP cells exhibit a lower cell growth after 5 days in 3D collagen matrices than shCtrl cells. This is linked with a higher apoptosis rate in cells silenced for MT1-MMP, as revealed in Figures 6D,E, which show an increase in both the number of annexin V positive cells and the mRNA level of the pro-apoptotic mediator BIK.

### DDR1 Overexpression in MDA-MB-231 Cells Increases Apoptosis

**Figure 7:**
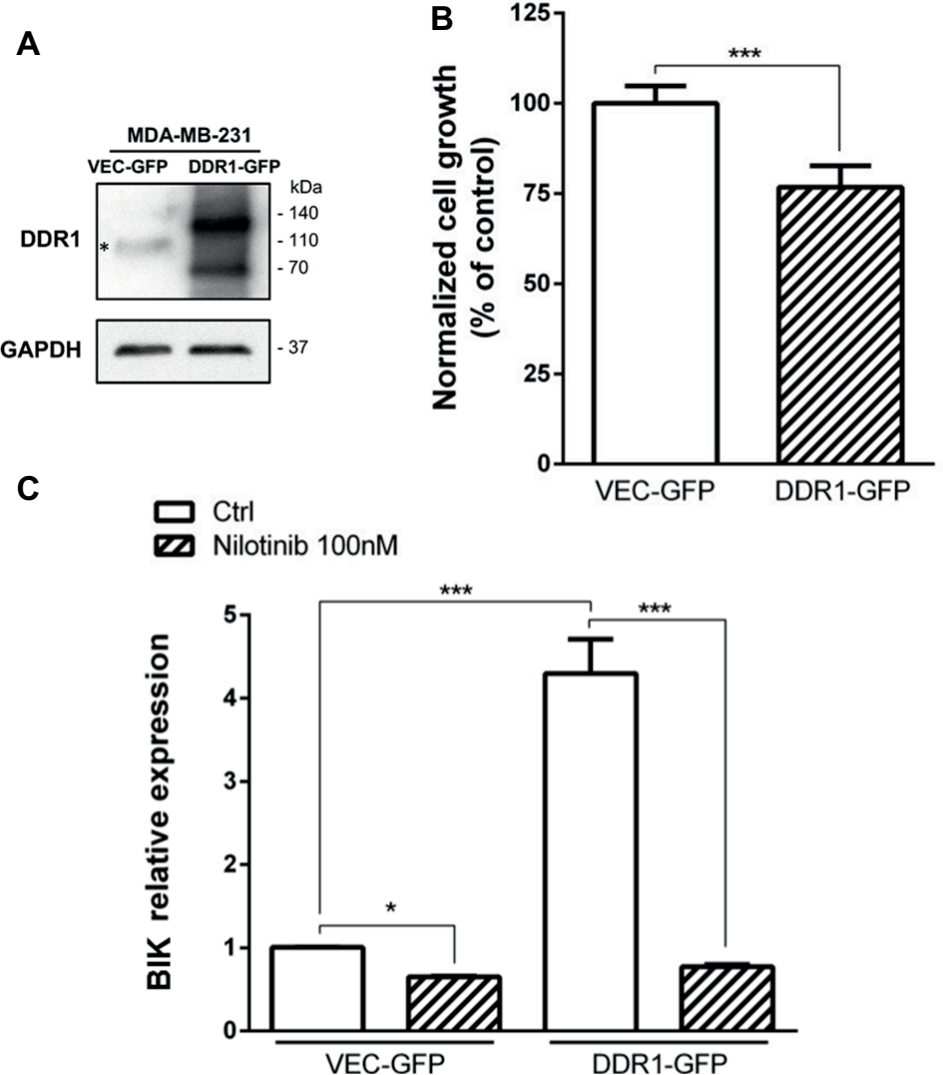
Effect of DDR1 expression in MDA-MB-231 on cell growth and survival in 3D type I collagen matrices. **(A)** Western blot analysis of ectopically expressed DDR1-GFP in MDA-MB-231. *: full-length endogenous DDR1. GAPDH was used as a loading control. **(B)** MDA-MB-231 VEC GFP (control GFP) and MDA-MB-231 DDR1-GFP cells were embedded in type I collagen 3D matrices. After 5 days of culture, viable cell density was evaluated by phase contrast microscopy. **(C)** MDA-MB-231 VEC GFP (control GFP) and MDA-MB-231 DDR1-GFP cells were embedded in 3D type I collagen matrices in absence (Ctrl) or in presence of nilotinib (100 nM). After 36 h of culture, BIK expression was measured by RT-PCR. Values represent the mean ± S.D. of three independent experiments (***p < 0.001).

We have previously shown that in MCF-7 cells, the decreased growth was induced by 3D type I collagen and was dependent on DDR1-induced apoptosis (Assent et al., 2015; Saby et al., 2018). Since MDA-MB-231 cells slightly express DDR1, we assume that the weak expression of this receptor is a key parameter explaining why type I collagen fails to impair growth and survival of these basal-like breast carcinoma cells. To address this hypothesis, MDA-MB-231 cells were transfected with a full-length DDR1-GFP expression vector (MDA-MB-231 DDR1-GFP) or an empty vector (MDA-MB-231 VEC-GFP), and cell growth and apoptosis were evaluated. As shown in Figure 7A, MDA-MB-231 DDR1-GFP expressed a high level of both full-length (140 kDa) and cleaved (70 kDa) DDR1-GFP, whereas MDA-MB-231 VEC-GFP only expresses low level of wild-type full length DDR1 (110 kDa). MDA-MB-231 DDR1-GFP and VEC-GFP were then seeded in 3D type I collagen matrices, and cell growth was quantified after 5 days. As shown in Figure 7B, DDR1 overexpression decreases MDA-MB-231 cell growth. Apoptosis was also evaluated by quantifying the expression of the proapoptotic BIK mRNA. As observed in Figure 7C, BIK is more expressed in MDA-MB-231 DDR1-GFP cells than in MDA-MB-231 VEC-GFP. Since DDR1 phosphorylation was affected by MT1-MMP expression status (Figure 6B), we measured the impact of nilotinib (100 nM), a receptor tyrosine kinase inhibitor with high potency against DDR1, on BIK expression. Figure 7C shows that nilotinib was able to significantly decrease BIK expression in both MDA-MB-231 VEC-GFP and MDA-MB-231 DDR1-GFP cells. Taken together, these data suggest that collagen-induced apoptosis is BIK dependent and relies on DDR1 phosphorylation.

### DDR1 Overexpression Coupled With MT1-MMP Depletion in MDA-MB-231 Cells Increases Cell Apoptosis

**Figure 8:**
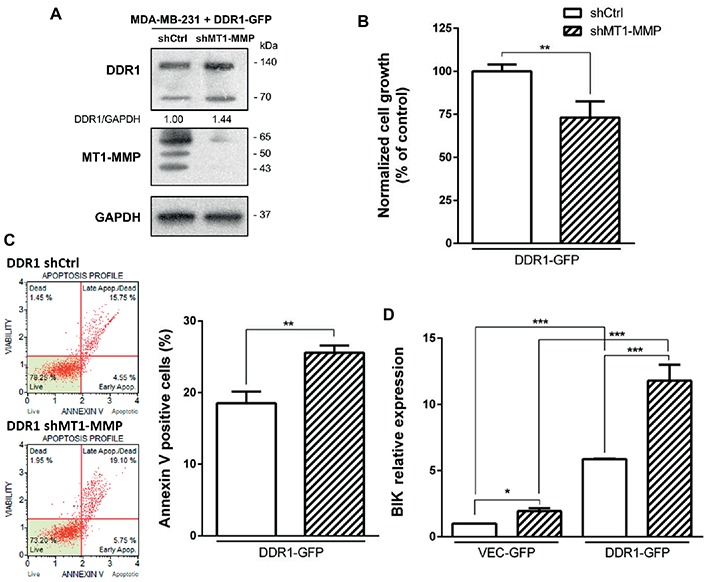
Effect of MT1-MMP silencing in MDA-MB-231 DDR1-GFP on cell growth and survival in 3D type I collagen matrices. **(A)** Western blot analysis confirming efficient knock down of MT1-MMP in MDA-MB-231 DDR1-GFP. GAPDH was used as a loading control. **(B,C)** MDA-MB-231 DDR1-GFP shCtrl and MDA-MB-231 DDR1-GFP shMT1-MMP cells were seeded in 3D type I collagen matrices. After 5 days of culture, viable cell density was evaluated by phase contrast microscopy **(B)**. After 36 h of culture, apoptosis was quantified using the Muse^®^ annexin V and dead cell assay kit **(C)**. **(D)** MDA-MB-231 DDR1-GFP shCtrl and MDA-MB-231 DDR1-GFP shMT1-MMP cells were embedded in 3D type I collagen matrices in absence (Ctrl) or in presence of nilotinib (100 nM). After 36 h of culture, BIK expression was measured by RT-PCR. Values represent the mean ± S.D. of three independent experiments (*p < 0.05, **p < 0.01, ***p < 0.001).

Since both DDR1 overexpression and MT1-MMP depletion were able individually to increase apoptosis in MDA-MB-231 cells, we hypothesized that the double transfection of DDR1 and shMT1-MMP in these cells could synergize to decrease cell growth and increase collagen-induced apoptosis to a level similar to that observed in luminal-like breast carcinoma cells. To this end, MDA-MB-231 cells overexpressing DDR1-GFP (MDA-MB-231 DDR1-GFP) were transfected with shRNA against MT1-MMP. As shown in Figure 8A, MT1-MMP depletion in these cells increases by 1.44-fold the expression of full length DDR1, when compared with MDA-MB-231 DDR1-GFP shCtrl cells. Then, cell growth and apoptosis in 3D collagen matrix were studied. As shown in Figure 8B, MDA-MB-231 DDR1-GFP exhibits a lower cell growth when MT1-MMP is depleted (MT1-MMP shRNA). This reduction is associated with a concomitant increase in apoptosis, as reflected by the increased number of annexin V positive cells (Figure 8C). We then studied the expression of the pro-apoptotic mediator BIK in those cells (Figure 8D). The data confirmed the previous results, with an increase in BIK mRNA expression in MDA-MB-231 DDR1-GFP treated with MT1-MMP shRNA compared to shCtrl. More importantly, Figure 8D showed that compared to MDA-MB-231 VEC-GFP, MDA-MB-231 DDR1-GFP cells exhibit about 6-fold increase in BIK mRNA level when MT1-MMP is depleted. Taken together, these data suggest that in basal-like breast carcinoma MDA-MB-231 cells, DDR1 activity and shMT1-MMP are able to synergize and increase collagen-induced apoptosis.

## Discussion

The extracellular matrix (ECM) represents an important component of the stromal microenvironment and functions as a master regulator of tumor behavior. In fact, the ECM plays key roles in major steps of cancer progression including cell proliferation, plasticity, survival and metastasis (Quail and Joyce, 2013; Pickup et al., 2014). Among ECM components, a complex network of adhesive proteins, including collagens, proteoglycans and glycoproteins, is able to regulate these different functions. These macromolecules are secreted by stromal cells and can be cleaved by several proteases including matrix metalloproteinases (MMPs), leading to the remodeling of the ECM (Bonnans et al., 2014).

The collagen superfamily is the major component of this ECM, particularly type I collagen which is the most abundant in several organs such as breast, lung, and skin (Mouw et al., 2014). In addition to its contribution to the architectural properties of the tissues, type I collagen is able to induce different cellular signaling pathways, which regulate several functions of tumor cells (Leitinger, 2011). Among type I collagen receptors, integrin heterodimers α1β1, α2β1, α10β1, and α11β1 are the most well known (Humphries et al., 2006). Recently, discoidin domain receptors have drawn special attention in the cross-talk between members of the collagen family, especially type I collagen and tumor cells. The particularity of these receptors is to be the only collagen receptors that possess a tyrosine kinase (RTK) activity. In addition, these receptors are characterized by a delayed and relatively long activation period (2 and 18 h, respectively) (Vogel et al., 1997; Fu et al., 2013b; Leitinger, 2014).

During tumor progression, especially after the degradation of the basement membrane, type I collagen is the major adhesive ECM protein encountered by invasive cancer cells, especially in breast carcinoma (Egeblad et al., 2010). We have previously shown that in 3D type I collagen matrix, DDR1 triggers a BIK-mediated apoptosis in poorly invasive luminal-like breast carcinomas cell lines (Assent et al., 2015; Saby et al., 2018). However, type I collagen failed to do so in the invasive basal-like MDA-MB-231 breast carcinoma cell line (Maquoi et al., 2012). This cell line is representative of aggressive breast cancer models, displaying a high metastatic ability associated with mesenchymal features. The expression of the membrane-anchored MT1-MMP, a major collagenolytic MMP, by the basal-like cancer cells has been largely described as a crucial step to promote the invasion process (Poincloux et al., 2009; Castro-Castro et al., 2016). Since the fibrillar organization of type I collagen is crucial for DDR1 activation (Vogel et al., 1997), the MT1-MMP-mediated degradation of type I collagen has been hypothesized to protect cancer cells from type I collagen-induced apoptosis. In accordance with this hypothesis, the enforced expression of MT1-MMP in luminal-like breast carcinoma cells abrogates the BIK-mediated apoptosis, suggesting that the degradation of type I collagen could be responsible of a lower DDR1 activation and thus a decreased apoptosis (Assent et al., 2015). A similar impairment of BIK-mediated apoptosis was also observed when these cells were exposed to aged type I collagen (Saby et al., 2018), which is characterized by an altered fibrillar organization (Aït-Belkacem et al., 2012).

However, MT1-MMP is also known to cleave DDR1, thereby reducing its activation (Fu et al., 2013a; Assent et al., 2015). As a consequence, MT1-MMP might also abrogate BIK-mediated apoptosis by negatively regulating the level of activated DDR1. To address this point and in order to study tumor cells harboring endogenous MT1-MMP, we investigated whether basal-like MDA-MB-231 breast carcinoma cells present a low level of DDR1 expression. As expected and at the opposite of the luminal-like MCF-7 breast cancer cells, MDA-MB-231 cells expressed MT1-MMP at a high level and DDR1 at a very low level (Figures 4A,B). Interestingly, we show that for Estrogen Receptor (ER) negative breast cancer patients, a low DDR1 expression is associated with a worse relapse-free survival (Figure 2B). It is worth noting that the mRNA level of the pro-apoptotic mediator BIK was positively correlated to that of DDR1 in human basal-like breast tumors (Figure 3). This suggests that tumors with high levels of DDR1 might be more sensitive to the BIK-mediated apoptotic pathway triggered by fibrillar type I collagen. It is important to state that the low level of DDR1 expression observed in most basal-like breast cancer cell lines is compensated by an upregulation of DDR2, with the noticeable exception of the MDA-MB-231 cells (Figure 1; Saby et al., 2018). This compensatory mechanism has been previously reported in invasive breast carcinoma from patients in which DDR1 and DDR2 were coordinately and inversely deregulated. In these patients, a DDR1 (Low)/DDR2 (High) expression profile has been associated with a significantly worse overall survival (Toy et al., 2015).

As shown in Figures 1, 4, DDR1 and MT1-MMP exhibit opposite expression patterns in MCF-7 and MDA-MB-231 cells. Previous works have reported that the DDR1 promoter region contains a p53 regulatory element and that wild-type p53 is able to induce DDR1 expression (Sakuma et al., 1996; Ongusaha et al., 2003). Interestingly, several studies that have investigated the mutational status of p53 in breast carcinoma have reported a wild type and mutated forms of p53 in MCF-7 and MDA-MB-231 cells, respectively (Lacroix et al., 2006; Muller and Vousden, 2014). Another possibility to explain this differential expression pattern of DDR1 between these two types of breast carcinoma could be related to a CpG methylation of the *DDR1* promoter during epithelial-mesenchymal transition (Chung et al., 2017).

Analysis of cell growth and apoptosis in MCF-7 cells showed that 3D collagen decreases cell growth by inducing apoptosis, whereas it failed to induce the same effects in MDA-MB-231 cells (Figures 4C,D). We then proposed to first verify whether enforced expression of MT1-MMP in MCF-7 cells was able to induce a protection against DDR1-mediated cell growth suppression and apoptosis. Accordingly, the enforced expression of MT1-MMP induced the cleavage of DDR1, thereby decreasing the abundance of the full length form of this receptor (Figures 5A,B). Interestingly, this cleavage was associated with an increase in cell growth and a decrease in apoptosis (Figures 5C,D).

We then investigated whether an endogenously expressed MT1-MMP is able to protect the basal-like MDA-MB-231 cells against BIK-mediated apoptosis and to regulate the level of DDR1. As shown in Figure 6, MT1-MMP depletion increased both full-length DDR1 expression and activation. As a direct consequence of this activation, the ability of type I collagen to suppress cell growth and to increase apoptosis was restored as evidenced by the increase in both annexin V staining and BIK expression (Figures 6C–E). This agrees with the positive correlation observed between DDR1 and BIK expression in human basal-like breast tumors (Figure 3). These results suggest that in MDA-MB-231 cells, the ability of type I collagen to suppress cell growth and to increase apoptosis is prevented by the conjunction of an intrinsically low level of DDR1 expression and a high MT1-MMP-dependent capacity to shed the extracellular domain of this receptor. Nevertheless, an association between a low or absent DDR1 expression with basal-like phenotype and worse relapse-free survival remains controversial. Recently, DDR1 knock-down in luminal-type MMTV-PyMT mammary tumor mouse model was shown to give rise to tumors displaying basal-type characteristics, a faster growth, and enhanced lung metastasis (Takai et al., 2018). This work suggested that DDR1 loss, by compromising cell adhesion and providing a cell growth advantage, contributes to the basal-like phenotype and consequently increases both the aggressiveness and metastatic potential of breast carcinomas. However, despite its low expression level in MDA-MB-231 cells, DDR1 was reported recently to induce the formation of linear invadosomes and to play an important role in proteolysis-based cell invasion in a collagen-rich environment (Juin et al., 2014; Moreau and Saltel, 2015; Di Martino et al., 2016). In this model, DDR1 depletion blocked cell invasion and, unexpectedly, its kinase activity was not required for invadosome formation (Juin et al., 2014).

We then investigated whether an increased DDR1 expression could sensitize the MDA-MB-231 cells toward the type I collagen-induced and BIK-mediated apoptosis. As shown in Figure 7C, enforced DDR1 expression significantly increased BIK expression in cells exposed to 3D collagen. This is in agreement with the positive correlation observed between DDR1 and BIK expression in basal-like tumors (Figure 3). To determine whether DDR1 kinase activity was required for this process, we treated MDA-MB-231 cells with its kinase function inhibitor nilotinib at a sub-toxic concentration. By using DDR1 and DDR2 gatekeeper mutations, we and others have previously demonstrated that nilotinib was able to inhibit specifically DDR1 and DDR2 (Beauchamp et al., 2014; Saby et al., 2016; Jeitany et al., 2018). By inhibiting DDR1 kinase function in MDA-MB-231 cells overexpressing DDR1, nilotinib was able to clearly prevent the upregulation of BIK mRNA (Figure 7C). Finally, in order to obtain expression levels of both DDR1 and MT1-MMP close to those observed in the luminal-like cell lines, DDR1 was overexpressed, and MT1-MMP was depleted in MDA-MB-231 cells. Under these settings, MT1-MMP depletion was able to further increase the expression of the full-length form of DDR1 (Figure 8A). This led to a decrease in cell growth that was associated with a significant increase in BIK-mediated apoptosis (Figures 8B,D). Collectively, these data reveal that both DDR1 and MT1-MMP represent key factors, in addition to the other mesenchymal markers, contributing to the aggressive phenotype in invasive breast carcinoma tumors.

**Figure 9:**
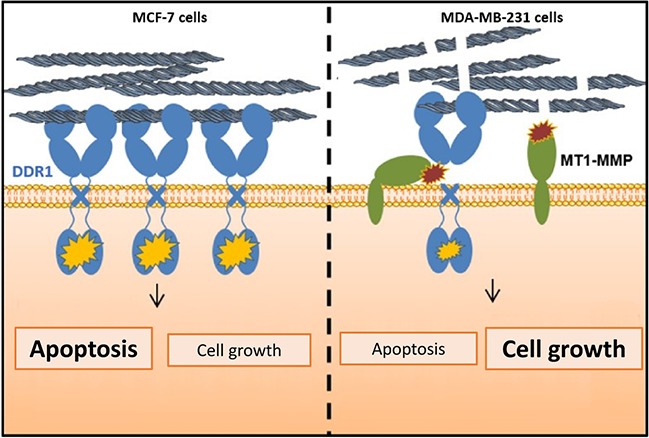
Proposed model highlighting the importance of MT1-MMP and DDR1 expression balance in the regulation of cell growth and survival in breast cancer cells cultivated in 3D type I collagen matrix.

In conclusion, and in agreement with the other studies (Ford et al., 2007; Koh et al., 2015; Toy et al., 2015; Takai et al., 2018), our data suggest that during the acquisition of mesenchymal features, the level of full-length DDR1 expression should be considered, in addition to the other markers, as an important biomarker in the prognosis of aggressive breast carcinomas. As summarized in Figure 9, the regulation of cell proliferation and apoptosis by the collagen/DDR1 axis involves a differential activation of DDR1 signaling pathway and thus BIK expression. In the case of the basal-like breast carcinoma cells, collagen proteolysis by MT1-MMP, the low expression of DDR1, and its cleavage by MT1-MMP could contribute to cell survival in the interstitial microenvironment. However, although our *in vitro* data suggest that low level of DDR1 expression should be considered as an additional important biomarker in the prognosis of breast carcinoma, these data need to be confirmed in future animal studies.

Finally, it is important to keep in mind that despite its low expression level in the aggressive breast carcinomas, DDR1 plays also a crucial role in the proteolysis-based cell invasion of a collagen-rich stroma (Juin et al., 2014).

## Author Contributions

All authors listed have made a substantial, direct and intellectual contribution to the work, and approved it for publication.

### Conflict of Interest Statement

The authors declare that the research was conducted in the absence of any commercial or financial relationships that could be construed as a potential conflict of interest.

## Footnotes

^1^www.cbioportal.org

^2^www.bcgenex.centregauducheau.fr
